# The impact of the muscle mass‐to‐fat ratio on the prognosis of patients undergoing pancreaticoduodenectomy for pancreatic cancer

**DOI:** 10.1002/kjm2.12928

**Published:** 2024-12-24

**Authors:** Long‐Jie Xu, Sheng‐Qiang Zhang, Chun Cao

**Affiliations:** ^1^ Department of General Surgery The Second Affiliated Hospital of Soochow University Suzhou China

**Keywords:** complications, muscle mass‐to‐fat ratio, overall survival, pancreatic cancer, pancreaticoduodenectomy

## Abstract

To evaluate the relationship between the muscle mass‐to‐fat ratio (MMFR) at the third lumbar spine (L3) and overall survival (OS) as well as related complications after pancreaticoduodenectomy (PD) for pancreatic cancer. Patients who underwent PD for pancreatic cancer between March 2017 and May 2023 at the Second Affiliated Hospital of Soochow University were included. Muscle mass and fat content at the L3 were measured by computed tomography. The specific formula that was used to calculate the MMFR was total abdominal muscle area/(subcutaneous adipose tissue area + visceral adipose tissue area), and the optimal cutoff values of the MMFR based on receiver operating characteristic curves were 0.688 for males and 0.382 for females. Patient characteristics were collected, and multivariate analyses were used to evaluate the impact of the MMFR on prognosis. Kaplan–Meier survival curves and log‐rank tests were used to compare OS between the high‐MMFR and low‐MMFR groups. On the basis of the optimal cutoff values, 191 patients were divided into two groups, with 91 patients in the low‐MMFR group and 100 patients in the high‐MMFR group. The incidence of POPF was significantly greater in the low‐MMFR group than in the high‐MMFR group. According to multivariate analysis, the MMFR was an independent factor associated with POPF and OS. Patients with low MMFRs had significantly shorter OS and a greater POPF incidence than did those with high MMFRs. The MMFR is an independent predictor of POPF and affects the OS of patients undergoing PD for pancreatic cancer.

## 1 INTRODUCTION

Considering its high mortality and morbidity rates and poor prognosis, pancreatic cancer is a fatal disease. Pancreatic cancer diagnoses have doubled worldwide in the past two decades.^1^ In addition, pancreatic cancer is the 7th leading cause of cancer‐related deaths worldwide, and the 5‐year survival rate is only 9%.^2^ Currently, various treatment options for pancreatic cancer are available, including preoperative neoadjuvant chemotherapy, postoperative adjuvant chemotherapy, and targeted therapy. However, despite its high recurrence rate, surgery remains the most effective treatment for pancreatic cancer.^3^ The main surgical method for treating pancreatic cancer, especially for malignant cancer of the pancreatic head, is pancreaticoduodenectomy (PD). Although comprehensive perioperative management has improved the overall survival (OS) and recurrence‐free survival of patients undergoing surgery and has made PD a safer procedure, this operation still has a high risk of morbidity and mortality.^4^ The major postoperative complications include postoperative pancreatic fistula (POPF), gastroplegia, bile leakage, chylous fistula, and intra‐abdominal abscess, and the occurrence of these complicates indicates a poor prognosis after surgery.^5^ In particular, POPF is the most common complication, the incidence of which reaches 29%.^6^ Patients who develop POPF are more likely to suffer from bleeding and intra‐abdominal infection.^7^ Therefore, accurate indicators that can be used to precisely evaluate surgical complications and OS are needed to help clinicians tailor treatment and improve long‐term survival.

Most recent studies have focused on the correlation between sarcopenia and the prognosis of malignant cancers. Sarcopenia has been reported to be associated with a poor prognosis in patients with lung cancer; these patients have a 4% increased risk of death for every unit of muscle mass lost.^8^ In addition, sarcopenia serves as a prognostic biomarker in patients with bladder cancer and gastric cancer and can lead to prolonged hospital stays, increased postoperative complications, and reduced quality of life.^9^ The progression‐free survival of patients with gastric cancer complicated by sarcopenia is significantly shorter than that of patients without sarcopenia.^10^ Among patients receiving palliative and curative treatment for pancreatic cancer, those with sarcopenia have poorer OS and shorter rates of disease‐free survival.^11^, ^12^ However, several studies suggest that fat mass is also essential for evaluating the prognosis of patients with malignant cancers. It has been reported that the visceral fat area is an independent predictor of OS in patients with epithelial ovarian, fallopian tube, or peritoneal cancer.^13^ A greater subcutaneous fat area is significantly associated with shorter OS in patients with gastric cancer.^14^ Skeletal muscle fat infiltration is an exact predictor of poor survival after surgery for gallbladder cancer and is a stronger predictor of survival than the quantity of skeletal muscle.^15^ A high ratio of the visceral fat area to the psoas muscle area is an independent prognostic factor for poor OS in patients after surgical resection of esophageal cancer.^16^ In addition, women who have a high fat‐to‐muscle ratio are more likely to develop breast cancer.^17^ Moreover, many studies have indicated that body composition plays a role in predicting the prognosis and OS of patients with pancreatic cancer. Several studies have emphasized the importance of decreasing muscle mass, which predicts poor OS after PD.^3^ An increased visceral fat tissue volume, as measured by computed tomography (CT), is also associated with the development of POPF after PD.^6^ The ratio of muscle to fat, as analyzed by bioelectrical impedance, is a predictor of cardiometabolic risk, such as coronary artery disease.^18^ However, it remains unclear whether the ratio of skeletal muscle mass to total fat, as measured at the third lumbar spine (L3) by CT, is a superior predictor of postoperative complications and OS in patients after PD.

In this study, we aimed to evaluate the relationship between the muscle mass‐to‐fat ratio (MMFR) at the L3 level and OS as well as related complications after PD in pancreatic cancer patients.

## 2 MATERIALS AND METHODS

### 2.1 Study population

A total of 200 patients who underwent PD from March 2017 to May 2023 at the Second Affiliated Hospital of Soochow University and who had a postoperative pathological diagnosis of pancreatic cancer were included in the study. Patients who did not undergo preoperative CT (9 patients) were excluded. Ultimately, a total of 191 patients were enrolled. Informed consent was obtained from all the patients, and the study was approved by the Ethics Committee of the Second Affiliated Hospital of Soochow University (JD‐HG‐2023‐0017).

### 2.2 CT image acquisition and management

**FIGURE 1:**
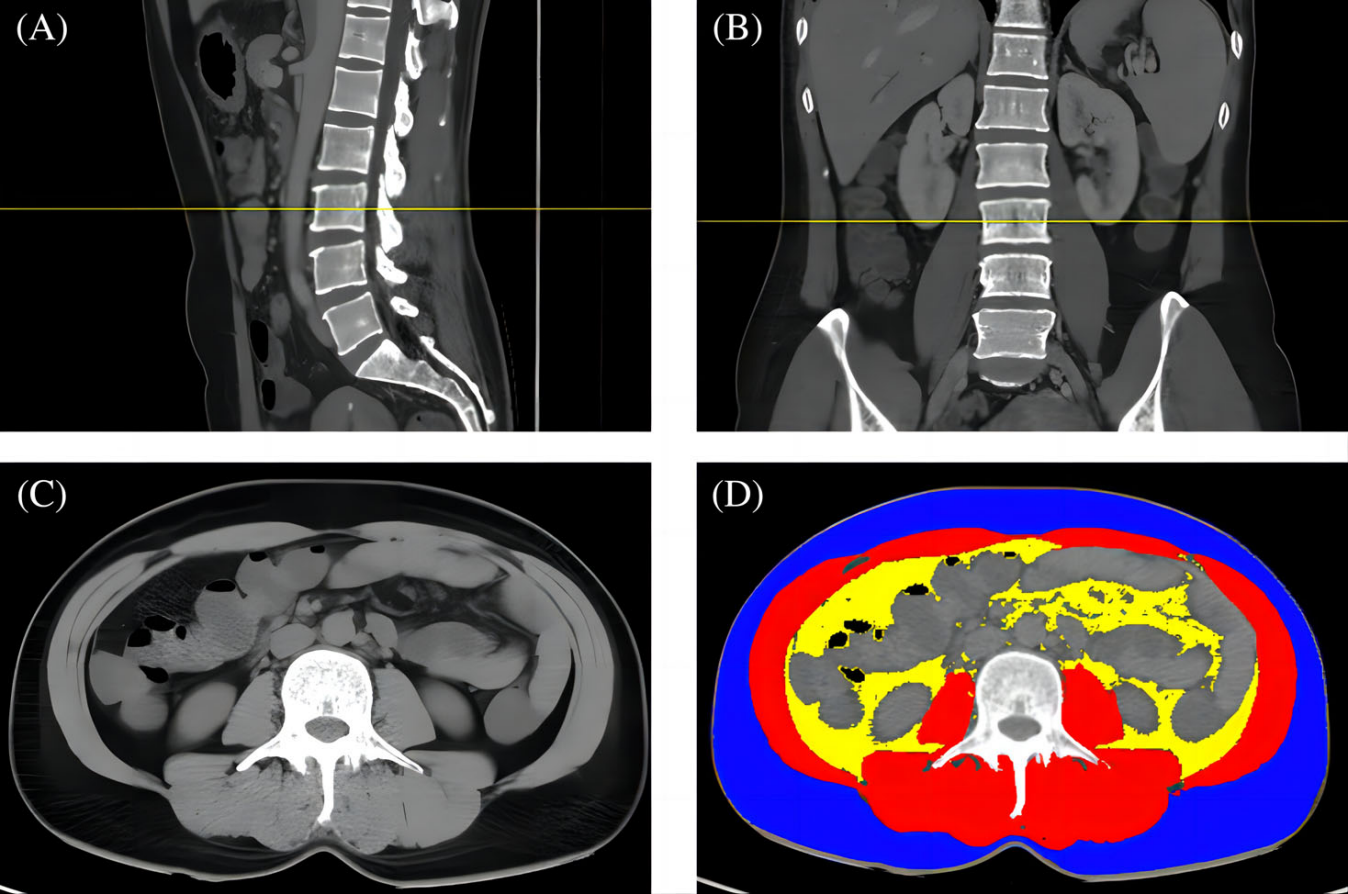
Computed tomography measurement of skeletal muscle area at the third lumbar vertebra (L3). (A) The middle segment of the third lumbar vertebra (L3) in the sagittal plane is marked with a yellow line; (B) The middle segment of L3 in the coronal plane is marked with a yellow line; (C) Horizontal cross section at L3; (D) TAMA was measured with a threshold from −29 to +150 HU (red); SAT was measured with a threshold from −190 to −30 HU (blue); and VAT was measured with a threshold from −150 to −50 HU (yellow). HU, Hounsfield unit; SAT, subcutaneous adipose tissue area; TAMA, total abdominal muscle area; VAT, visceral adipose tissue area.

After locating the position of L3, cross‐sectional images were collected for all patients. Each image was analyzed via SliceOmatic software 5.0 (TomoVision, USA) according to the standard Hounsfield Unit (HU) range. The subcutaneous adipose tissue (SAT) area at −190 to −30 HU, the visceral adipose tissue (VAT) area at −150 to −50 HU, and the total abdominal muscle (TAMA) area at −29 to 150 HU were determined. The body composition of the participants is shown in Figure 1.

### 2.3 Patient characteristics

The patients' background and preoperative characteristics included sex, age, body mass index (BMI), visceral‐to‐subcutaneous adipose tissue area ratio (VSR), sarcopenia status, preoperative serious underlying disease, and preoperative laboratory examination (conducted 2 days before surgery). Intraoperative characteristics included operation time, amount of bleeding, main pancreatic duct (MPD) diameter, anastomosis of pancreatojejunostomy method, vascular reconstruction, pancreatic texture (defined as soft or hard according to the intraoperative judgment of the operating surgeons), blood transfusion, vascular invasion, nerve invasion, and lymphatic invasion. Postoperative characteristics included intensive care unit (ICU) admission status, hospital stay, postoperative hospital stay, hospitalization expenses, tumor‐lymph node‐metastasis (TNM) stage, postoperative laboratory examination on postoperative day (POD) 3, mortality, survival time, and overall complications, such as POPF, bile leakage, chylous fistula, gastrointestinalgia, sepsis, hemorrhage, organ space surgical site infection (SSI), incisional SSI, intestinal obstruction, reoperation, thrombotic events, pneumonia, cardiopulmonary events, and Clavien–Dindo classification (CDC).

### 2.4 Definition of body composition

**FIGURE 2:**
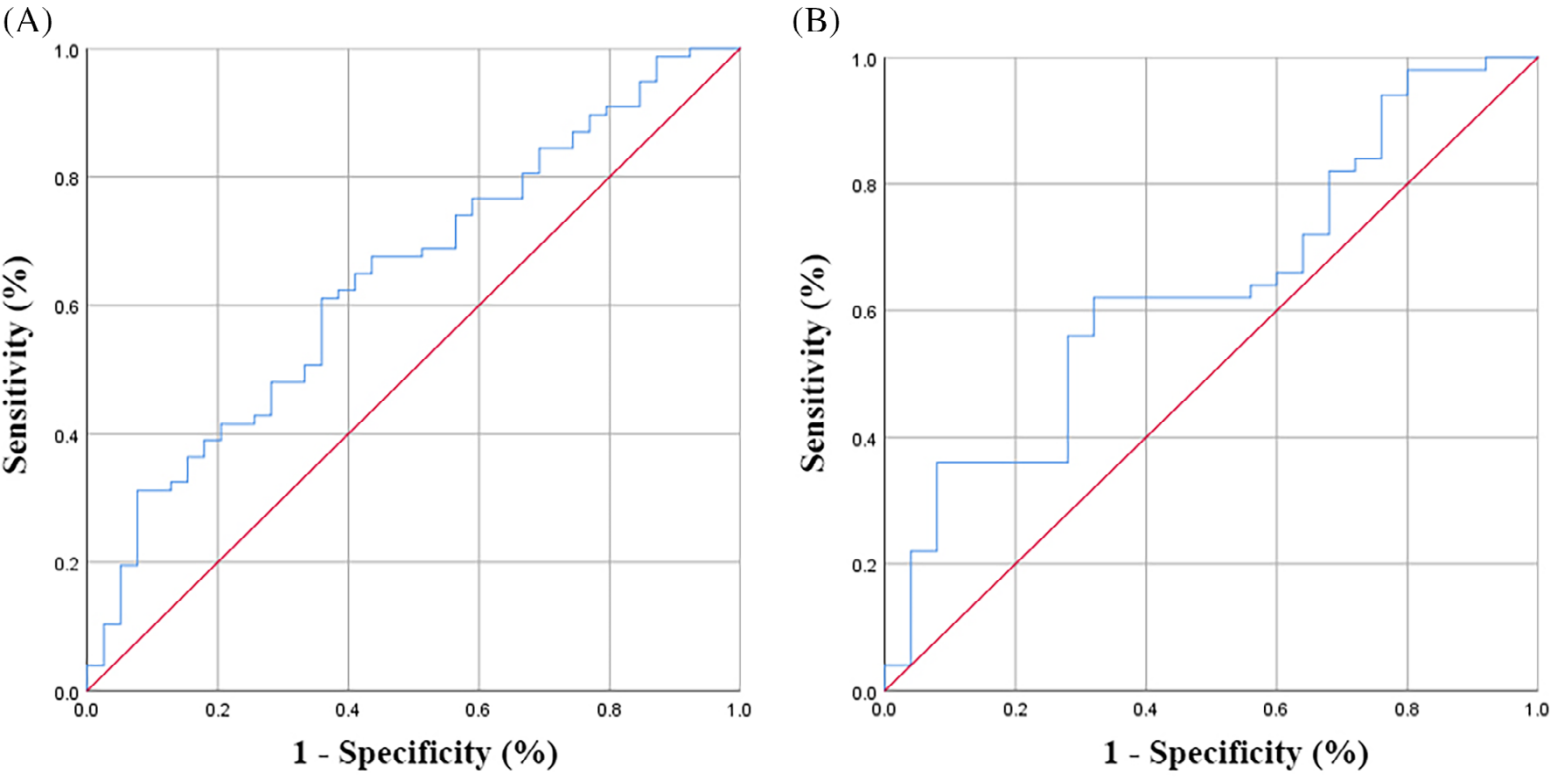
Receiver operating characteristic curve for the MMFR stratified by sex. (A) male, the area under the curve = 0.643; sensitivity =61.0%; specificity = 64.1%. (B) female, the area under the curve = 0.636; sensitivity = 62.0%; specificity = 68.0%.

The specific formula for calculating the MMFR was TAMA/(SAT + VAT), which was based on our previous study,^19^ and the optimal cutoff values of the MMFR based on receiver operating characteristic (ROC) curves were 0.688 for males and 0.382 for females (Figure 2). Sarcopenia was defined as a skeletal muscle index (SMI) <41 cm^2^/m^2^ in females and <43 cm^2^/m^2^ in males with a BMI <25 kg/m^2^ and <53 cm^2^/m^2^ in males with a BMI >25 kg/m^2^. The VSR refers to the ratio of VAT to SAT, and the optimal cutoff value of the VSR based on the ROC curves was 0.486.

### 2.5 Statistical analyses

Continuous variables are expressed as medians and interquartile ranges (IQRs). The Kolmogorov–Smirnov test was used to test the normality of the data. Normally distributed variables were analyzed by Student's *t* test, and nonnormally distributed variables were analyzed by the nonparametric Mann–Whitney *U* test. Categorical variables are expressed as numbers and percentages and were analyzed by the chi‐square test or Fisher's exact test. The factors that were significantly different (*p* < 0.1) according to univariate logistic regression were included in the multivariate analysis. The Cox proportional hazards model was used for survival analysis, and all factors that were significantly correlated (*p* < 0.1) with OS were entered into the multivariate analysis. The results are reported as the adjusted odds ratio (OR) with the corresponding 95% confidence interval (CI). Survival curves were analyzed via Kaplan–Meier curves and compared via the log‐rank test. All tests were two‐sided, and *p* < 0.05 was used as an indicator of statistical significance. All the statistical analyses were performed via SPSS software 26.0 (SPSS, Chicago, IL, USA).

## 3 RESULTS

Among the 191 patients enrolled, 116 (60.7%) were male, the median age was 68 years (IQR: 60–73 years), and the BMI was 22.2 kg/m^2^ (IQR: 20.3–24.5 kg/m^2^). Ninety‐one patients met the criteria for inclusion in the low‐MMFR group, and 100 patients met the criteria for inclusion in the high‐MMFR group according to the optimal cutoff values stratified by sex.

### 3.1 Baseline characteristics according to the MMFR

There were significant differences between the high‐MMFR and low‐MMFR groups in terms of preoperative baseline characteristics, including age (*p* = 0.042), BMI (*p* < 0.001), VSR (*p* < 0.001), hemoglobin (Hb) (*p* = 0.001), globulin (*p* = 0.033), and fibrinogen (*p* = 0.048). However, sex, sarcopenia status, preoperative serious underlying disease status, and other preoperative laboratory data were not significantly different between the two groups (Table 1).

**TABLE 1:** Patient demographic and clinical characteristics according to the MMFR.

Variable	High MMFR (*n* = 100)	Low MMFR (*n* = 91)	*p* value
Sex			
Male	61 (61.0%)	55 (60.4%)	0.937
Female	39 (39.0%)	36 (39.6%)	
Age (years)	66 (57, 73)	69 (63, 74)	0.042*
BMI (kg/m^2^)	20.95 (19.29, 22.83)	24.00 (22.04, 25.61)	<0.001*
VSR	0.737 (0.474, 1.032)	1.121 (0.770, 1.481)	<0.001*
Sarcopenia			
Yes	52 (52.0%)	48 (52.7%)	0.918
No	48 (48.0%)	43 (47.3%)	
Preoperative serious underlying disease			
Yes	6 (6.0%)	2 (2.2%)	0.283
No	94 (94.0%)	89 (97.8%)	
Preoperative laboratory examination			
WBC (× 10^9^/L)	5.9 (4.3, 7.6)	5.8 (4.7, 7.3)	0.701
Lymphocyte (× 10^9^/L)	1.4 (1.0, 1.6)	1.3 (1.1, 1.7)	0.654
Neutrophil (× 10^9^/L)	3.6 (2.5, 5.1)	3.8 (2.7, 5.0)	0.478
Platelet (× 10^9^/L)	240 (171, 284)	236 (198, 295)	0.517
Hb (g/L)	117 (107, 129)	126 (113, 138)	0.001*
CRP (mg/L)	5.5 (5.1, 7.2)	5.5 (4.8, 6.4)	0.624
Albumin (g/L)	39.0 (36.4, 43.0)	40.4 (37.4, 44.7)	0.050
Globulin (g/L)	27.0 (24.1, 29.6)	27.9 (26.2, 31.4)	0.033*
Albumin/Globulin	1.43 (1.25, 1.67)	1.43 (1.26, 1.60)	0.527
Prealbumin (g/L)	0.21 (0.15, 0.25)	0.18 (0.13, 0.24)	0.208
INR	1.00 (0.95, 1.06)	1.00 (0.95, 1.05)	0.755
APTT (s)	36.3 (33.1, 39.6)	35.0 (32.8, 38.8)	0.268
PT (s)	13.0 (12.6, 13.7)	13.1 (12.6, 13.6)	0.913
Fibrinogen (mg/dL)	3.62 (2.89, 4.55)	3.92 (3.19, 4.81)	0.048*
TBil (μmol/L)	28.9 (10.0, 103.2)	17.7 (10.8, 72.5)	0.495
ALT (U/L)	52 (17, 88)	40 (16, 109)	0.855
AST (U/L)	37 (20, 63)	29 (17, 78)	0.642
ALP (U/L)	201 (85, 437)	123 (82, 336)	0.158
LDH (U/L)	172 (148, 195)	174 (156, 206)	0.267
GGT (U/L)	198 (33, 533)	142 (29, 479)	0.653

Abbreviations: ALP, alkaline phosphatase; ALT, alanine aminotransferase; APTT, activated partial thromboplastin time; AST, aspartate aminotransferase; BMI, body mass index; CRP, C‐reactive protein; GGT, gamma glutamyl transferase; Hb, hemoglobin; INR, international normalized ratio; LDH, lactate dehydrogenase; MMFR, muscle mass‐to‐fat ratio; PT, prothrombin time; TBil, total bilirubin; VSR, visceral‐to‐subcutaneous adipose tissue area ratio; WBC, white blood cell.

* *p* < 0.05.

### 3.2 Intraoperative characteristics according to the MMFR

As shown in Table 2, the pancreatic texture significantly differed between the low‐MMFR and high‐MMFR groups (*p* = 0.004). However, other intraoperative characteristics, including the operation time, amount of bleeding, MPD diameter, anastomosis of pancreatojejunostomy method, vascular reconstruction, blood transfusion, vascular invasion, nerve invasion, and lymphatic invasion, were not different between the two groups.

**TABLE 2:** Intraoperative characteristics according to the MMFR.

Variable	High MMFR (*n* = 100)	Low MMFR (*n* = 91)	*p* value
Operation time (min)	320 (300, 360)	345 (300, 395)	0.104
Amount of bleeding (mL)	300 (163, 400)	300 (200, 500)	0.213
MPD diameter (mm)			
<3 mm	36 (36.0%)	42 (46.2%)	0.154
≥3 mm	64 (64.0%)	49 (53.8%)	
Methods of anastomosis			
Continuous suture	55 (55.0%)	47 (51.6%)	0.643
“8‐character” suture	45 (45.0%)	44 (48.4%)	
Vascular reconstruction			
Yes	1 (1.0%)	3 (3.3%)	0.548
No	99 (99.0%)	88 (96.7%)	
Pancreatic texture			
Soft	26 (26.0%)	42 (46.2%)	0.004*
Hard	74 (74.0%)	49 (53.8%)	
Blood transfusion			
Yes	26 (26.0%)	32 (35.2%)	0.169
No	74 (74.0%)	59 (64.8%)	
Vascular invasion			
Yes	25 (25.0%)	22 (24.2%)	0.895
No	75 (75.0%)	69 (75.8%)	
Nerve invasion			
Yes	42 (42.0%)	36 (39.6%)	0.732
No	58 (58.0%)	55 (60.4%)	
Lymphatic invasion			
Yes	29 (29.0%)	24 (26.4%)	0.686
No	71 (71.0%)	67 (73.6%)	

Abbreviations: MMFR, muscle mass‐to‐fat ratio; MPD, main pancreatic duct.

* *p* < 0.05.

### 3.3 Postoperative complications and characteristics according to the MMFR

The overall complication rate was significantly different between the high‐MMFR and low‐MMFR groups (*p* = 0.012). In particular, the incidences of POPF, bile leakage, and organ space SSI were significantly lower in the high‐MMFR group than in the low‐MMFR group (*p* < 0.001; *p* = 0.043; *p* = 0.045, respectively). However, other complications, including chylous fistula, gastroplegia, sepsis, hemorrhage, incisional SSI, intestinal obstruction, thrombotic events, pneumonia, and cardiopulmonary events, were not different between the two groups. In addition, there were no significant differences between the two groups in terms of reoperation, ICU admission status, hospital stay, postoperative hospital stay, hospitalization expense, or TNM stage. The levels of neutrophils, C‐reactive protein (CRP), fibrinogen, and lactate dehydrogenase on POD 3 were significantly lower in the high‐MMFR group than in the low‐MMFR group (*p* = 0.030; *p* = 0.002; *p* = 0.024; *p* = 0.008, respectively). In addition, the levels of albumin and prealbumin were significantly greater in the high‐MMFR group than in the low‐MMFR group (*p* = 0.005; *p* < 0.001, respectively). The other postoperative laboratory examinations revealed no significant differences (Table 3).

**TABLE 3:** Postoperative complications and characteristics according to the MMFR.

Variable	High MMFR (*n* = 100)	Low MMFR (*n* = 91)	*p* Value
Overall complications			
Yes	60 (60.0%)	70 (76.9%)	0.012*
No	40 (40.0%)	21 (23.1%)	
POPF			
Yes	22 (22.0%)	42 (46.2%)	<0.001*
No	78 (78.0%)	49 (53.8%)	
Bile leakage			
Yes	12 (12.0%)	21 (23.1%)	0.043*
No	88 (88.0%)	70 (76.9%)	
Chylous fistula			
Yes	4 (4.0%)	7 (7.7%)	0.274
No	96 (96.0%)	84 (92.3%)	
Gastrointestinalgia			
Yes	30 (30.0%)	35 (38.5%)	0.218
No	70 (70.0%)	56 (61.5%)	
Sepsis			
Yes	7 (7.0%)	5 (5.5%)	0.668
No	93 (93.0%)	86 (94.5%)	
Hemorrhage			
Yes	9 (9.0%)	12 (13.2%)	0.356
No	91 (91.0%)	79 (86.8%)	
Organ space SSI			
Yes	24 (24.0%)	34 (37.4%)	0.045*
No	76 (76.0%)	57 (62.6%)	
Incisional SSI			
Yes	4 (4.0%)	5 (5.5%)	0.626
No	96 (96.0%)	86 (94.5%)	
Intestinal obstruction			
Yes	4 (4.0%)	7 (7.7%)	0.274
No	96 (96.0%)	84 (92.3%)	
Thrombotic events			
Yes	3 (3.0%)	4 (4.4%)	0.711
No	97 (97.0%)	87 (95.6%)	
Pneumonia			
Yes	13 (13.0%)	17 (18.7%)	0.281
No	87 (87.0%)	74 (81.3%)	
Cardiopulmonary events			
Yes	7 (7.0%)	7 (7.7%)	0.855
No	93 (93.0%)	84 (92.3%)	
CDC ≥ III			
Yes	37 (37.0%)	46 (50.5%)	0.059
No	63 (63.0%)	45 (49.5%)	
Reoperation			
Yes	7 (7.0%)	10 (11.0%)	0.344
No	93 (93.0%)	81 (89.0%)	
ICU admission status			
Yes	71 (71.0%)	64 (70.3%)	0.919
No	29 (29.0%)	27 (29.7%)	
Hospital stays (days)	32 (26, 39)	32 (26, 43)	0.511
Postoperative hospital stays (days)	23 (18, 26)	23 (20, 33)	0.133
Hospitalization expenses (×10^4^ RMB)	11.40 (8.95, 13.67)	10.88 (9.52, 14.86)	0.363
TNM stage			
III	5 (5.0%)	6 (6.6%)	0.637
I–II	95 (95.0%)	85 (93.4%)	
Postoperative laboratory examination			
WBC (× 10^9^/L)	10.6 (7.9, 14.0)	11.8 (8.8, 16.1)	0.071
Lymphocyte (× 10^9^/L)	0.8 (0.7, 1.0)	0.8 (0.6, 1.0)	0.326
Neutrophil (× 10^9^/L)	8.9 (6.5, 11.7)	10.2 (7.3, 14.0)	0.030*
Platelet (× 10^9^/L)	193 (144, 240)	182 (140, 229)	0.516
Hb (g/L)	98 (90, 108)	99 (87, 108)	0.774
CRP (mg/L)	95.4 (56.0, 145.8)	124.9 (77.0, 181.1)	0.002*
Albumin (g/L)	35.1 (31.8, 37.7)	32.9 (30.8, 35.8)	0.005*
Globulin (g/L)	21.7 (18.7, 24.4)	21.1 (18.6, 23.6)	0.621
Albumin/Globulin	1.64 (1.43, 1.96)	1.59 (1.36, 1.78)	0.162
Prealbumin (g/L)	0.11 (0.09, 0.15)	0.1 (0.07, 0.12)	<0.001*
INR	1.123 (1.06, 1.21)	1.13 (1.04, 1.23)	0.875
APTT (s)	37.1 (31.2, 43.2)	38.9 (32.5, 43.8)	0.442
PT (s)	14.2 (13.1, 14.9)	14.1 (13.1, 15.2)	0.680
Fibrinogen (mg/dL)	4.5 (3.3, 5.5)	5.1 (3.7, 5.9)	0.024*
TBil (μmol/L)	26.6 (11.7, 63.5)	22.3 (11.9, 42.9)	0.839
ALT (U/L)	31 (20, 47)	30 (17, 48)	0.792
AST (U/L)	23 (16, 33)	23 (16, 30)	0.731
ALP (U/L)	106 (73, 197)	100 (58, 153)	0.110
LDH (U/L)	183 (156, 211)	200 (169, 241)	0.008*
GGT (U/L)	104 (39, 194)	67 (31, 172)	0.319

Abbreviations: ALP, alkaline phosphatase; ALT, alanine aminotransferase; APTT, activated partial thromboplastin time; AST, aspartate aminotransferase; CDC, Clavien–Dindo classification; CRP, C‐reactive protein; GGT, gamma glutamyl transferase; Hb, hemoglobin; ICU, intensive care unit; INR, international normalized ratio; LDH, lactate dehydrogenase; MMFR, muscle mass‐to‐fat ratio; POPF, postoperative pancreatic fistula; PT, prothrombin time; SSI, surgical site infection; TBil, total bilirubin; TNM, tumor‐lymph node‐metastasis; WBC, white blood cell.

* *p* < 0.05.

### 3.4 Univariate and multivariate logistic regression analyses of risk factors for POPF

As shown in Table 4, the VSR, MPD diameter, MMFR, pancreatic texture, lymphatic invasion, preoperative white blood cell count, CRP, albumin/globulin, alanine aminotransferase, and alkaline phosphatase concentrations were determined via univariate analysis. Multivariate logistic regression analysis confirmed that MPD diameter (OR, 10.76; 95% CI, 4.666–24.83; *p* = 0.001), MMFR (OR, 2.894; 95% CI, 1.214–6.899; *p* = 0.017), lymphatic invasion (OR, 4.590; 95% CI, 1.599–13.17; *p* = 0.005), and CRP (OR, 2.943; 95% CI, 1.061–8.158; *p* = 0.038) were independent predictors of POPF.

**TABLE 4:** Univariate and multivariate logistic regression analyses of risk factors for POPF.

Variable	Univariate analysis		Multivariate analysis	
	OR (95% CI)	*p* Value	OR (95% CI)	*p* Value
Sex (female vs. male)	0.987 (0.533, 1.830)	0.967		
Age > 65 ys (Yes vs. No)	0.677 (0.365, 1.260)	0.217		
BMI < 18 kg/m^2^ (Yes vs. No)	0.309 (0.067, 1.425)	0.132		
VSR <0.486 (Yes vs. No)	0.272 (0.090, 0.819)	0.021*	0.558 (0.136, 2.287)	0.417
MPD diameter <3 mm (Yes vs. No)	9.700 (4.825, 19.50)	0.001*	10.76 (4.666, 24.83)	0.001*
Methods of anastomosis (continuous suture vs. “8‐character” suture)	1.307 (0.713, 2.396)	0.386		
Vascular reconstruction (Yes vs. No)	0.656 (0.067, 6.436)	0.718		
Sarcopenia (Yes vs. No)	0.790 (0.433, 1.441)	0.442		
MMFR (Low vs. High)	3.039 (1.623, 5.692)	0.001*	2.894 (1.214, 6.899)	0.017*
TNM stage (III vs. I‐II)	1.709 (0.501, 5.829)	0.392		
ICU admission status (Yes vs. No)	1.225 (0.627, 2.396)	0.553		
Preoperative serious underlying disease (Yes vs. No)	2.050 (0.496, 8.480)	0.322		
Operation time > 330 min (Yes vs. No)	1.116 (0.611, 2.036)	0.722		
Amount of bleeding >300 mL (Yes vs. No)	0.632 (0.345, 1.157)	0.137		
Pancreatic texture (Soft vs. Hard)	2.570 (1.371, 4.816)	0.003*	1.852 (0.801, 4.280)	0.150
Blood transfusion (Yes vs. No)	0.851 (0.439, 1.649)	0.633		
Vascular Invasion (Yes vs. No)	0.697 (0.338, 1.439)	0.329		
Nerve Invasion (Yes vs. No)	0.600 (0.320, 1.124)	0.111		
Lymphatic invasion (Yes vs. No)	4.624 (1.948, 10.98)	0.001*	4.590 (1.599, 13.17)	0.005*
Preoperative laboratory examination				
WBC ≥9 × 10^9^/L (Yes vs. No)	2.721 (1.064, 6.957)	0.037*	3.182 (0.827, 12.25)	0.092
Lymphocyte ≤ 1.1 × 10^9^/L (Yes vs. No)	1.531 (0.823, 2.846)	0.178		
Neutrophil ≥ 6 × 10^9^/L (Yes vs. No)	1.321 (0.538, 3.240)	0.543		
Platelet ≤ 125 × 10^9^/L (Yes vs. No)	0.843 (0.211, 3.375)	0.809		
Hb ≤ 100 g/L (Yes vs. No)	1.495 (0.624, 3.582)	0.367		
CRP ≥ 10 mg/L (Yes vs. No)	1.935 (0.931, 4.023)	0.077*	2.943 (1.061, 8.158)	0.038*
Albumin < 32 g/L (Yes vs. No)	1.143 (0.322, 4.057)	0.836		
Globulin < 25 g/L (Yes vs. No)	1.078 (0.536, 2.167)	0.834		
Albumin/Globulin < 1.5 (Yes vs. No)	1.874 (1.019, 3.447)	0.043*	2.241 (0.948, 5.300)	0.066
Prealbumin < 0.2 g/L (Yes vs. No)	0.896 (0.491, 1.635)	0.720		
APTT > 45 s (Yes vs. No)	2.033 (0.399, 10.37)	0.393		
PT > 14 s (Yes vs. No)	1.343 (0.588, 3.068)	0.484		
Fibrinogen < 2 mg/dL (Yes vs. No)	0.488 (0.053, 4.459)	0.525		
TBil > 100 μmol/L (Yes vs. No)	0.631 (0.301, 1.324)	0.223		
ALT > 60 U/L (Yes vs. No)	1.752 (0.923, 3.327)	0.087*	1.253 (0.458, 3.433)	0.660
AST > 45 U/L (Yes vs. No)	0.700 (0.370, 1.324)	0.273		
ALP > 100 U/L (Yes vs. No)	2.067 (1.111, 3.844)	0.022*	1.310 (0.475, 3.614)	0.602
LDH > 250 U/L (Yes vs. No)	0.703 (0.215, 2.302)	0.560		
GGT > 60 U/L (Yes vs. No)	0.722 (0.388, 1.344)	0.304		

*Note*: Univariate logistic regression analysis: *p < 0.1; multivariate logistic regression analysis: *p < 0.05.

Abbreviations: ALP, alkaline phosphatase; ALT, alanine aminotransferase; APTT, activated partial thromboplastin time; AST, aspartate aminotransferase; BMI, body mass index; CRP, C‐reactive protein; GGT, gamma glutamyl transferase; Hb, hemoglobin; ICU, intensive care unit; INR, international normalized ratio; LDH, lactate dehydrogenase; MMFR, muscle mass‐to‐fat ratio; MPD, main pancreatic duct; POPF, postoperative pancreatic fistula; PT, prothrombin time; TBil, total bilirubin; TNM, tumor‐lymph node‐metastasis; VSR, visceral‐to‐subcutaneous adipose tissue area ratio; WBC, white blood cell.

### 3.5 OS analysis

**FIGURE 3:**
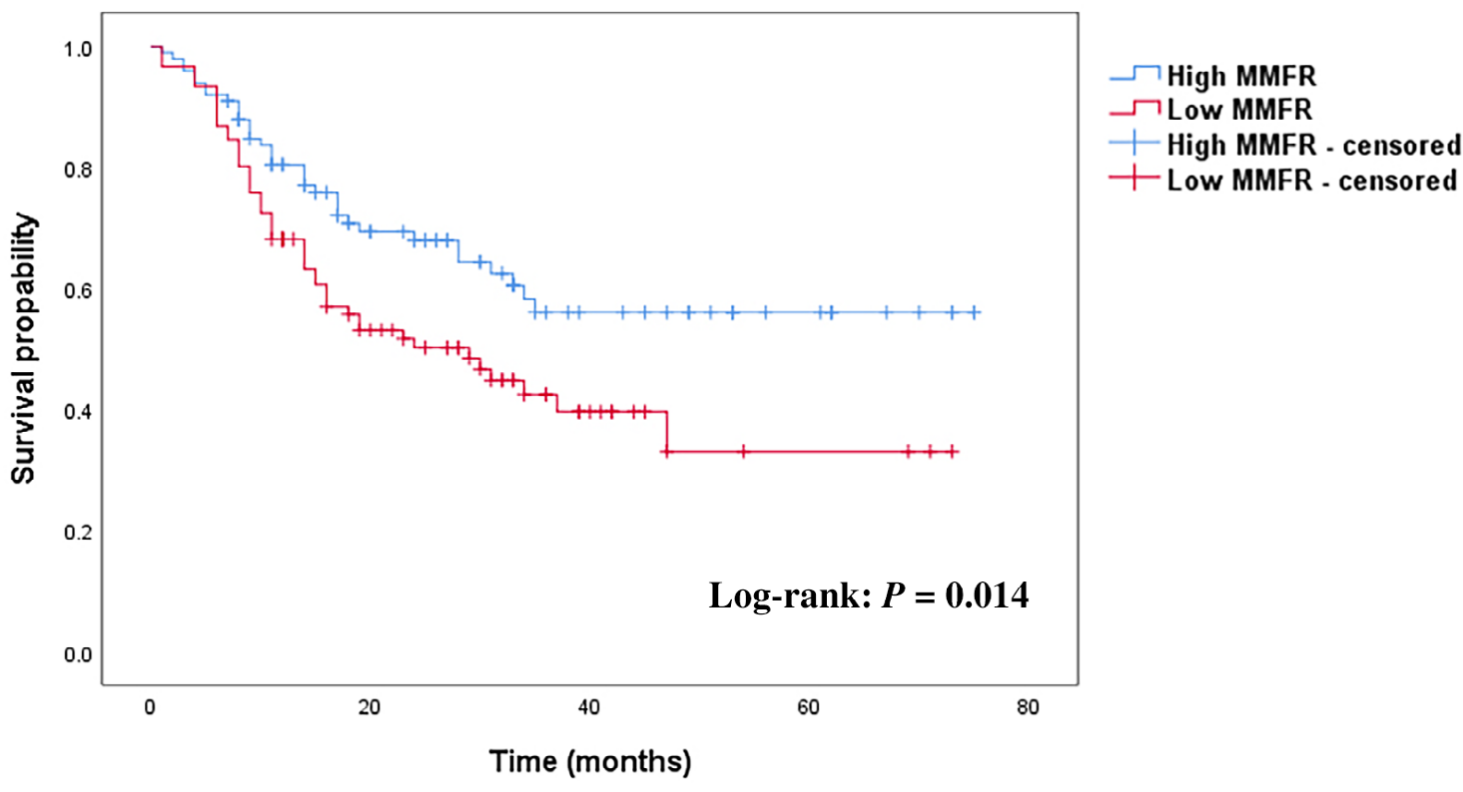
Overall survival according to muscle mass‐to‐fat ratio. MMFR, muscle mass‐to‐fat ratio.

The results of univariate and multivariate Cox regression analyses of OS among patients who underwent PD for pancreatic cancer are shown in Table 5. According to the univariate analysis, age, vascular reconstruction, sarcopenia, MMFR, ICU admission status, blood transfusion, nerve invasion, lymphatic invasion, sepsis, and cardiopulmonary events were significant predictors of OS after surgery. Multivariate analysis revealed that vascular reconstruction (HR, 4.120; 95% CI, 1.235–13.75; *p* = 0.021), sarcopenia (HR, 1.778; 95% CI, 1.124–2.811; *p* = 0.014), MMFR (HR, 1.718; 95% CI, 1.084–2.723; *p* = 0.021), ICU admission status (HR, 1.907; 95% CI, 1.078–3.373; *p* = 0.026), and nerve invasion (HR, 1.734; 95% CI, 1.101–2.730; *p* = 0.017) were independent predictors of OS. Moreover, the OS curves revealed that patients with low MMFRs had significantly shorter OS than did those with high MMFRs (*p* = 0.014) (Figure 3).

**TABLE 5:** Univariate and multivariate Cox regression analyses of risk factors for overall survival.

Variable	Univariate analysis		Multivariate analysis	
	HR (95% CI)	*p* Value	HR (95% CI)	*p* Value
Sex (female vs. male)	0.983 (0.635, 1.520)	0.938		
Age > 65 (Yes vs. No)	2.027 (1.243, 3.306)	0.005*	1.159 (0.674, 1.993)	0.594
BMI < 18 kg/m^2^ (Yes vs. No)	0.774 (0.313, 1.913)	0.579		
VSR <0.486 (Yes vs. No)	0.750 (0.398, 1.415)	0.374		
MPD diameter <3 mm (Yes vs. No)	1.255 (0.803, 1.961)	0.319		
Methods of anastomosis (Continuous suture vs. “8‐character” suture)	0.863 (0.562, 1.325)	0.499		
Vascular reconstruction (Yes vs. No)	3.674 (1.140, 11.84)	0.029*	4.120 (1.235, 13.75)	0.021*
Sarcopenia (Yes vs. No)	1.779 (1.144, 2.768)	0.011*	1.778 (1.124, 2.811)	0.014*
MMFR (Low vs. High)	1.705 (1.104, 2.633)	0.016*	1.718 (1.084, 2.723)	0.021*
TNM stage (III vs. I‐II)	1.359 (0.626, 2.948)	0.438		
ICU admission status (Yes vs. No)	2.048 (1.187, 3.533)	0.010*	1.907 (1.078, 3.373)	0.026*
Preoperative serious underlying disease (Yes vs. No)	0.565 (0.139, 2.298)	0.425		
Operation time > 330 min (Yes vs. No)	1.177 (0.766, 1.808)	0.456		
Amount of bleeding >300 mL (Yes vs. No)	1.426 (0.915, 2.223)	0.117		
Pancreatic texture (Soft vs. Hard)	1.267 (0.819, 1.960)	0.287		
Blood transfusion (Yes vs. No)	1.579 (1.019, 2.448)	0.041*	1.114 (0.690, 1.799)	0.658
Vascular invasion (Yes vs. No)	1.465 (0.910, 2.357)	0.116		
Nerve invasion (Yes vs. No)	1.879 (1.222, 2.888)	0.004*	1.734 (1.101, 2.730)	0.017*
Lymphatic invasion (Yes vs. No)	1.608 (1.031, 2.508)	0.036*	1.511 (0.952, 2.398)	0.080
Overall complications (Yes vs. No)	1.013 (0.638, 1.610)	0.955		
POPF (Yes vs. No)	0.739 (0.454, 1.202)	0.222		
Bile leakage (Yes vs. No)	1.473 (0.852, 2.546)	0.166		
Chylous fistula (Yes vs. No)	0.599 (0.189 1.897)	0.383		
Gastrointestinalgia (Yes vs. No)	1.390 (0.899, 2.150)	0.139		
Sepsis (Yes vs. No)	1.971 (0.949, 4.092)	0.069*	1.809 (0.822, 3.980)	0.141
Hemorrhage (Yes vs. No)	0.972 (0.486, 1.941)	0.935		
Organ space SSI (Yes vs. No)	1.072 (0.675, 1.702)	0.770		
Incisional SSI (Yes vs. No)	1.349 (0.544, 3.341)	0.518		
Intestinal obstruction (Yes vs. No)	1.248 (0.544, 2.864)	0.601		
Reoperation (Yes vs. No)	1.601 (0.801, 3.200)	0.183		
Thrombotic events (Yes vs. No)	1.657 (0.607, 4.528)	0.325		
Pneumonia (Yes vs. No)	1.219 (0.686, 2.164)	0.500		
Cardiopulmonary events (Yes vs. No)	1.864 (0.933, 3.726)	0.078*	1.791 (0.843, 3.808)	0.130
CDC ≥ III (Yes vs. No)	1.104 (0.719, 1.696)	0.650		
Postoperative laboratory examination				
WBC ≥9 × 10^9^/L (Yes vs. No)	1.194 (0.728, 1.956)	0.482		
Lymphocyte ≤1.1 × 10^9^/L (Yes vs. No)	1.154 (0.638, 2.084)	0.636		
Neutrophil ≥6 × 10^9^/L (Yes vs. No)	0.749 (0.421, 1.331)	0.325		
Platelet ≤125 × 10^9^/L (Yes vs. No)	1.140 (0.652, 1.994)	0.646		
Hb ≤100 g/L (Yes vs. No)	1.369 (0.880, 2.128)	0.163		
CRP ≥10 mg/L (Yes vs. No)	20.31 (0, 5.8 × 10^6)	0.639		
Albumin <32 g/L (Yes vs. No)	1.092 (0.698, 1.706)	0.701		
Globulin <25 g/L (Yes vs. No)	1.584 (0.839, 2.989)	0.156		
Albumin/Globulin <1.5 (Yes vs. No)	0.773 (0.494, 1.210)	0.260		
Prealbumin <0.2 g/L (Yes vs. No)	0.951 (0.347, 2.602)	0.922		
APTT >45 s (Yes vs. No)	1.375 (0.832, 2.272)	0.214		
PT > 14 s (Yes vs. No)	0.965 (0.629, 1.482)	0.872		
Fibrinogen <2 mg/dL (Yes vs. No)	1.076 (0.340, 3.410)	0.901		
TBil >100 μmol/L (Yes vs. No)	1.418 (0.811, 2.480)	0.220		
ALT >60 U/L (Yes vs. No)	1.024 (0.555, 1.890)	0.939		
AST > 45 U/L (Yes vs. No)	0.642 (0.260, 1.586)	0.337		
ALP > 100 U/L (Yes vs. No)	1.409 (0.915, 2.170)	0.119		
LDH > 250 U/L (Yes vs. No)	0.957 (0.519, 1.763)	0.887		
GGT > 60 U/L (Yes vs. No)	1.465 (0.927, 2.317)	0.102		

Abbreviations: ALP, alkaline phosphatase; ALT, alanine aminotransferase; APTT, activated partial thromboplastin time; AST, aspartate aminotransferase; BMI, body mass index; CDC, Clavien–Dindo classification; CRP, C‐reactive protein; GGT, gamma glutamyl transferase; Hb, hemoglobin; ICU, intensive care unit; INR, international normalized ratio; LDH, lactate dehydrogenase; MMFR, muscle mass‐to‐fat ratio; MPD, main pancreatic duct; POPF, postoperative pancreatic fistula; PT, prothrombin time; SSI, surgical site infection; TBil, total bilirubin; TNM, tumor‐lymph node‐metastasis; VSR, visceral‐to‐subcutaneous adipose tissue area ratio; WBC, white blood cell.

* *p* < 0.05.

## 4 DISCUSSION

In this study, the MMFR was identified as an independent predictor of OS by both univariate and multivariate Cox regression analyses. In addition, multivariate logistic regression analysis identified the MMFR as an independent prognostic factor for POPF in patients with pancreatic cancer. These results indicated a strong correlation between the preoperative MMFR and the prognosis of patients after PD, suggesting that adding the MMFR could aid in the critical optimization of prognostic prediction models for pancreatic cancer.

Pancreatic cancer, characterized by inapparent early symptoms and difficult diagnosis, is a malignant cancer with a high mortality rate and poor prognosis. Surgical treatment has been the only radical treatment for pancreatic cancer, but its poor prognosis has been a major problem. Recently, many studies have explored the relationship between body composition and postoperative prognosis in PD patients, and they have shown that muscle mass and fat components are associated with the prognosis of PD.^20^, ^21^, ^22^ According to our study, the level of postoperative albumin was significantly lower in patients with low MMFRs than in patients with high MMFRs; low postoperative albumin levels predict susceptibility to malnutrition and increase the risk of delayed anastomotic and incisional healing and abdominal infection in patients with low MMFRs. Recent studies have shown that nutritional support can regulate innate immunity and inflammation, in turn promoting anastomotic healing and reducing the incidence of postoperative complications.^23^ In contrast, malnutrition can reduce collagen synthesis and granuloma formation, which leads to delayed anastomotic healing and creates a favorable environment for bacterial growth, thus increasing the risk of infection. In addition, low serum albumin can lead to tissue edema by reducing the osmotic pressure of plasma colloids and promoting the leakage of tissue interstitial fluid into the wound, providing a medium for bacterial growth and further increasing the risk of infectious complications.^24^ Patients in the low‐MMFR group had significantly more neutrophils and CRP after PD, suggesting that patients with a low MMFR have severe postoperative inflammation. Recent studies have shown that visceral fat can produce adipokines, such as leptin, resistin, and adiponectin, which regulate inflammation and metabolism. Imbalances in these adipokines, particularly elevated levels of leptin, can contribute to inflammation.^25^ In addition, visceral fat promotes the accumulation of macrophages in fat tissue by increasing local extracellular lipid concentrations, and macrophages release proinflammatory cytokines such as tumor necrosis factor‐alpha and interleukin‐6,^25^ which further exacerbates the inflammatory response. Moreover, excessive visceral fat and less abdominal muscle could cause immune response dysregulation,^26^ which could also contribute to severe inflammation. And we found that the VSR is greater in patients with a low MMFR. These studies may explain why patients with low MMFRs have a greater incidence of organ space SSI.

Patients in the high‐MMFR group were less likely to experience overall complications than those in the low‐MMFR group. POPF is the most common complication after PD, and the incidence was 33.5% in this study, which is similar to the incidence reported in a recent study.^27^ Sarcopenia has been reported to be an independent risk factor for POPF,^21^ and visceral fat can also independently predict POPF.^28^ Furthermore, the ratio of visceral fat to skeletal muscle has been shown to independently predict POPF.^29^ Our study revealed that the MMFR is a significant predictor of POPF rather than sarcopenia, and patients with a low MMFR are more likely to experience POPF. Several mechanisms may explain why patients with low MMFRs are more likely to develop POPF after PD. First, excessive abdominal fat can increase the difficulty of accessing the pancreas during surgery, possibly leading to inadvertent injury to the pancreatic duct or incomplete closure of the pancreatic stump, thereby increasing the risk of POPF. Second, excessive fatty infiltration of the pancreas and soft pancreatic texture lead to pancreatic hypertrophy and a soft pancreatic texture, which results in incomplete anastomosis. Third, excessive abdominal fat and low muscle mass are associated with compromised blood supply to adipose tissues, including the pancreas. Insufficient blood flow to pancreatic tissues can impair wound healing and predispose patients to the development of POPF. Moreover, excessive adipose tissue leads to the production and secretion of adipocytokines such as leptin, tumor necrosis factor‐α, interleukin (IL)‐1 and IL‐6, which can suppress the immune system and delay wound healing, thereby increasing the risk of POPF.^21^ In addition, a study indicated that the MPD diameter is smaller in patients with POPF than in patients without POPF.^30^ In our study, MPD diameter was an independent predictor of POPF, possibly because a greater MPD diameter facilitates the anastomosis of pancreatojejunostomy, thus reducing the development of POPF. Our study revealed that postoperative CRP is an independent predictor of POPF. Elevated CRP usually indicates acute inflammation.^31^ One study revealed a positive correlation between systemic inflammatory response syndrome and clinically relevant POPF.^32^ Thus, CRP may contribute to POPF by activating systemic inflammatory response syndrome.

Our study revealed that the MMFR is an independent predictor of OS in patients after PD and that OS is significantly lower in patients with low MMFR than in those with high MMFR. The high incidence of postoperative complications in patients in the low‐MMFR group increased the length of bed rest, abdominal drain placement, and parenteral nutrition, which delays the initiation of postoperative functional exercise and contributes to the poor OS of patients after PD. In addition, our study revealed that postoperative albumin was lower in patients with low MMFRs than in those with high MMFRs, suggesting poorer postoperative nutritional status in the low‐MMFR group than in the high‐MMFR group. A recent study showed that malnutrition could increase chemotherapeutic toxicity by altering the distribution, metabolism, and clearance of systemic chemotherapeutic agents.^33^ Malnutrition can also weaken the immune system, increase susceptibility to infections and delay recovery from chemotherapy‐induced side effects, which can result in dose reductions or delays in treatment, thereby compromising its effectiveness.^33^ Moreover, malnourished patients have decreased liver function and reduced physical endurance,^34^ which increases the difficulty of eliminating chemotherapy drugs and tolerating the side effects of chemotherapy. As a result, these drugs may accumulate at relatively high levels in the body, leading to increased toxicity and a greater risk of side effects. In addition, decreased skeletal muscle mass has been shown to contribute to lower doses of chemotherapy and intolerance of chemotherapy side effects by causing frailty, impairing physical function, reducing quality of life, and increasing toxicity during chemotherapy, ultimately leading to reduced efficacy of the drugs.^35^ Excessive visceral fat increases the volume of drug distributed via the accumulation of drugs in fat tissue and alters the pharmacokinetics of chemotherapeutic agents by influencing hepatic and renal drug clearance, both of which result in decreased efficacy and increased toxicity of chemotherapeutic agents.^36^ Considering that most patients after PD undergo postoperative adjuvant chemotherapy, which is significantly associated with OS, the mechanisms described above may explain the relationship between low MMFRs and poor OS. One study indicated that sarcopenia is associated with OS but is not associated with postoperative complications,^11^ which is compatible with our study. In addition, our study revealed that vascular reconstruction and nerve invasion are independent risk factors for OS. Vascular reconstruction increases the difficulty of the surgical procedure, prolongs the operating time, and increases the risk of intraoperative hemorrhage, which has a negative impact on OS. Furthermore, patients with nerve invasion may be less sensitive to chemotherapy and radiotherapy, and cancer cells invading along nerves may undergo a number of biological changes that make them resistant to conventional chemotherapeutic agents and radiotherapy,^37^ both of which might contribute to a short OS.

Our study has several strengths. First, we included only patients with pancreatic cancer who underwent PD, which eliminated the impact of different pathologies and surgical methods on OS. Second, preoperative CT is a routine imaging method for patients with pancreatic cancer, and body composition can be obtained via preoperative CT, which allows patients to avoid additional examinations and costs. This study has several limitations. Because this was a retrospective study involving patients from a single center, there may be selection bias. Therefore, multicenter studies with larger sample sizes are needed to further validate our results.

In conclusion, preoperative muscle mass combined with fat content independently predicted POPF and OS in patients who underwent PD for pancreatic cancer. Patients with a low MMFR had a significantly greater risk of poor prognosis. A low MMFR is often associated with increased inflammation and malnutrition, which suggests that comprehensive anti‐inflammatory therapy and nutritional support are warranted for patients with a low MMFR.

## CONFLICT OF INTEREST STATEMENT

The authors declare no conflict of interest.
